# Development of Repetitive Mechanical Oscillation Needle-Free Injection through Electrically Induced Microbubbles

**DOI:** 10.34133/cbsystems.0225

**Published:** 2025-03-19

**Authors:** Yibo Ma, Wenjing Huang, Naotomo Tottori, Yoko Yamanishi

**Affiliations:** ^1^ Bio-medical Fluid Engineering Laboratory, Mechanical Engineering, Kyushu University, Fukuoka, Japan.; ^2^ Kindai University Technical College, Mie, Japan.

## Abstract

We previously developed a novel needle-free reagent injection method based on electrically induced microbubbles. The system generates microbubbles and applies repetitive mechanical oscillation associated with microbubble dynamics to perforate tissue and introduce a reagent. In this paper, we propose improving the reagent injection depth by reflecting the shock wave through microbubble dynamics. Our results show that the developed shock wave reflection method improves the ability of the electrically induced microbubble injection system to introduce a reagent. The method extends the application potential of electrically induced microbubble needle-free injection.

## Introduction

Currently, drug administration for disease treatment and prophylaxis generally adopts an injector with a metal needle. Drug delivery using a metallic needle is a useful technique because it allows easy skin penetration and controlled drug delivery. However, because the needle is in direct contact with the patient’s mucus and blood, the spread of infectious diseases through the use of different syringes has long been a worldwide problem [1]. To solve this problem, needle-free drug injection systems have been developed worldwide [2–4]. Most needle-free drug injection systems perforate the skin and introduce a drug through a high-pressure water jet [4]. The high-pressure water jet can be generated using any of a large number of methods. Commercial needle-free injectors use compressed air, a loaded spring, piezo actuators, or electrical discharge as the power source to generate the high-pressure water jet [5,6]. Taberner et al. [7] developed a system using an injector with a Lorenz-force motor, Kyriazis et al. [8] designed a system using shaped charge, and Tagawa et al. [9,10] developed a system using a laser introducing system to generate the high-pressure water jet. Despite the widespread development of water-jet needle-free injectors, the application of jet injector techniques is limited by reduced flexibility, an uncontrolled fluid stream, and cross-contamination. Recently, researchers have proposed and developed a new method for needle-free drug injection. Kislov et al. [11] introduced a novel needle-free injection system based on the abnormal optothermal expansion of mesoporous vaterite cargoes. They accelerated the vaterite particles using infrared laser illumination and successfully delivered them into a phantom layer and Xenopus oocytes in vitro, demonstrating a new concept for needle-free injection. Krizek et al. [12] developed a novel needle-free injection method based on a laser-induced jet. They generated the jet through optical cavitation triggered by a laser pulse. The laser was delivered via multimode optical fiber, and their system is expected to be integrated into minimally invasive surgical procedures.

We have developed a novel micro-scale tool, referred to as an electrically induced microbubble generator, and developed it for many applications, such like cell DNA transfection [13] and the metallization of elastomers [14,15]. Furthermore, we have developed the electrically induced microbubble generator for needle-free injection through repetitive mechanical oscillation due to microbubble dynamics. The system has potential advantages over existing needle-free injection technologies. Comparing to the existing needle-free injectors with a 1- to 10-mm-diameter tip [4,16,17], the diameter of the microbubble generator tip was approximately 0.1 mm. This gives it the flexibility to be integrated into a wide range of surgical devices as a compact minimally invasive device. Because our method utilizes microjets and shock waves in a liquid, it is expected to deliver both liquid and soluble drugs to the target. Moreover, comparing to the current laser-induced cavitation needle-free injection systems [9,18,19] that utilize a laser power source, the power source of our method was the power source of electric scalpel that is a common device in hospitals. The tip of our device was an electrode. It is relatively inexpensive ($5 for one tip) [14] and replaceable. During clinical application, it is easy for the operator to learn. This makes our method have the potential to be used by clinical medicine in large quantities.

In this research, we improved the reagent introduction ability of an electrically induced microbubble needle-free injection system by reflecting the shock wave from the microbubble dynamics. We compared the injection depth with and without the shock wave reflection and imaged the shock wave through schlieren photography. Our results show that the use of a shock-wave reflection device could achieve the reflection of shock wave and improved the reagent depth by approximately 200 μm.

### Concept

#### Electrically induced microbubble

**Fig. 1.**
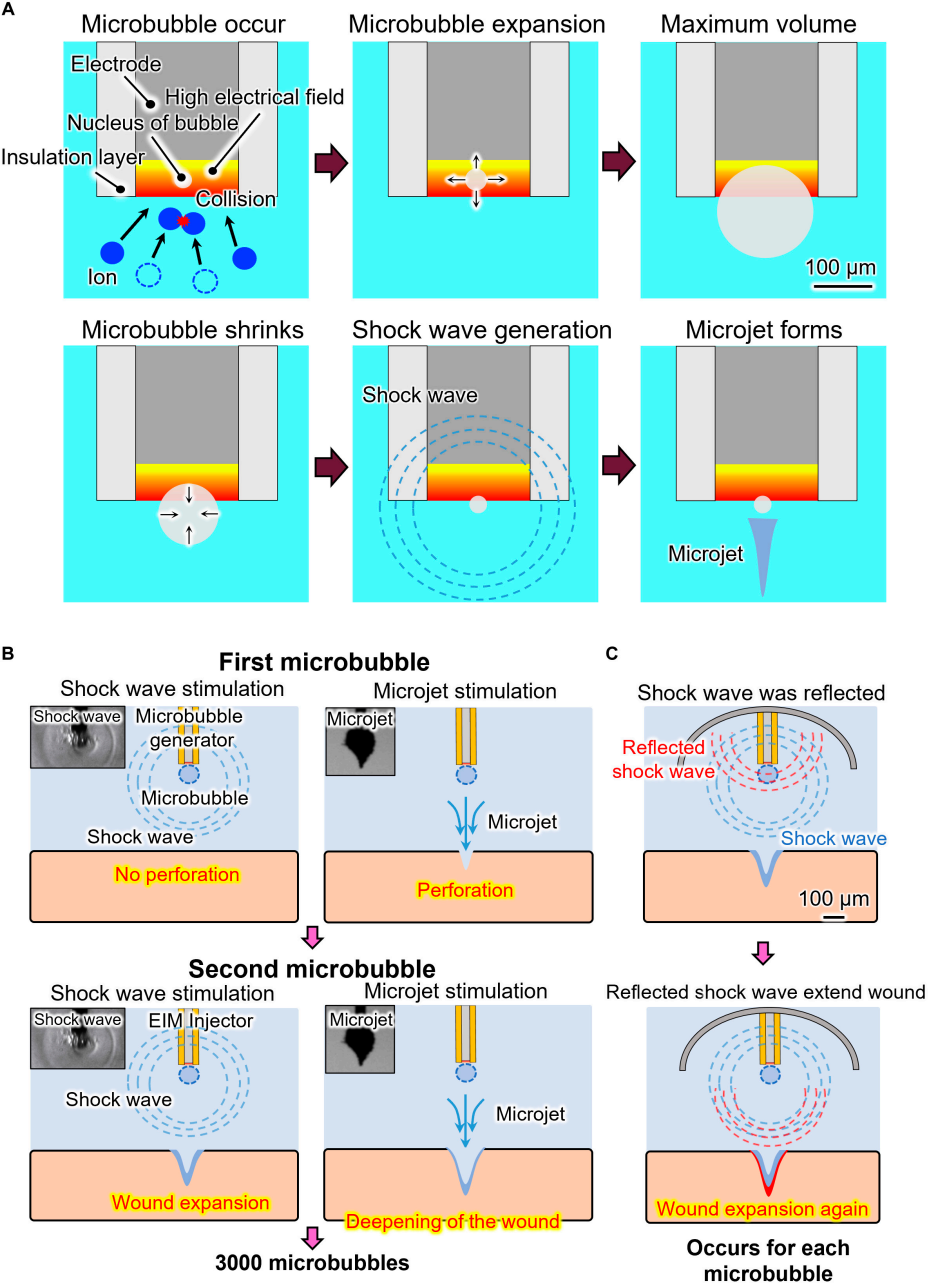
Concept of repetitive mechanical oscillation needle-free injection based on electrically induced microbubbles. (A) Dynamics of the electrically induced microbubble, showing the formation, expansion, and shrinking of the microbubble and the shock wave and microjet from the microbubble. (B) Mechanism of the microbubble repetitive mechanical oscillation injection, microjet perforation of the target, shock wave expanding the wound, and the repeated process. (C) Mechanism of shock wave reflection enhancing perforation.

Cavitation in the liquid can be generated through the optothermal effect, laser induction, high voltage application, etc. [11,20]. The electrically induced microbubble injector generates microbubbles by applying a high voltage to an electrode [21]. The microbubble dynamics are shown in Fig. 1A. When the high-voltage electric field is concentrated at the tip of the injector, the ions near the tip move at high speed and collide with each other owing to the high-voltage electric field. The collision of ions disperses molecules and eventually leads to the production of tiny bubbles [21–23]. When the generated electric field exceeds the dielectric constant of the bubbles, a plasma and discharge are generated [24]. The temperature within a tiny bubble rises rapidly, and the pressure of the bubble is not released in the process [25–28]. The thermoelastic stress generated in this process is confined inside the tiny bubble, which leads to a maximum pressure rise inside the bubble [29]. The tiny bubble then expands rapidly until reaching a maximum volume. The microbubble starts to shrink after reaching the maximum volume. A shock wave is generated when the microbubble collapses to a minimum volume. A microjet then forms. The dynamics of an electrically induced microbubble are shown in Fig. 1A. A typical video showing an electrically induced microbubble from its generation to collapse is provided in Movie S1.

#### Microbubble repetitive mechanical oscillation needle-free injection

The concept of tissue injection using electrically induced microbubbles is shown in Fig. 1. When a voltage pulse is applied to an electrode, there is a concentrated high-voltage electric field at the electrode tip. A microbubble forms at the electrode tip. The microbubble expands and collapses. As the microbubble collapses, a shock wave is generated and a microjet forms. At the first voltage pulse, the shock wave is transmitted to the tissue and the tissue vibrates without perforation. The microjet then forms and perforates the tissue. At the second voltage pulse, the shock wave is transmitted to the tissue and the perforation wound expands through the vibration of the tissue. The microjet then perforates and deepens the wound. This process was repeated for 3,000 cycles to achieve tissue injection. The mechanism of microbubble repetitive mechanical oscillation needle-free injection is shown in Fig. 1B.

#### Shock wave reflection enhancing perforation

We propose a semi-ellipsoid reflector to reflect the shock wave from a microbubble. The mechanism of shock wave reflection is shown in Fig. 1C. The shock wave is transmitted in all directions after the microbubble collapses. Only the part of the shock wave transmitted toward the target expands the wound, and most of the mechanical energy of the shock wave is thus lost. However, part of the shock wave transmitted away from the target is reflected by the reflector and thus transmitted to the target. The reflected shock wave expands the wound again. This process occurs at each microbubble generation and improves the perforation ability.

## Materials and Methods

**Fig. 2.**
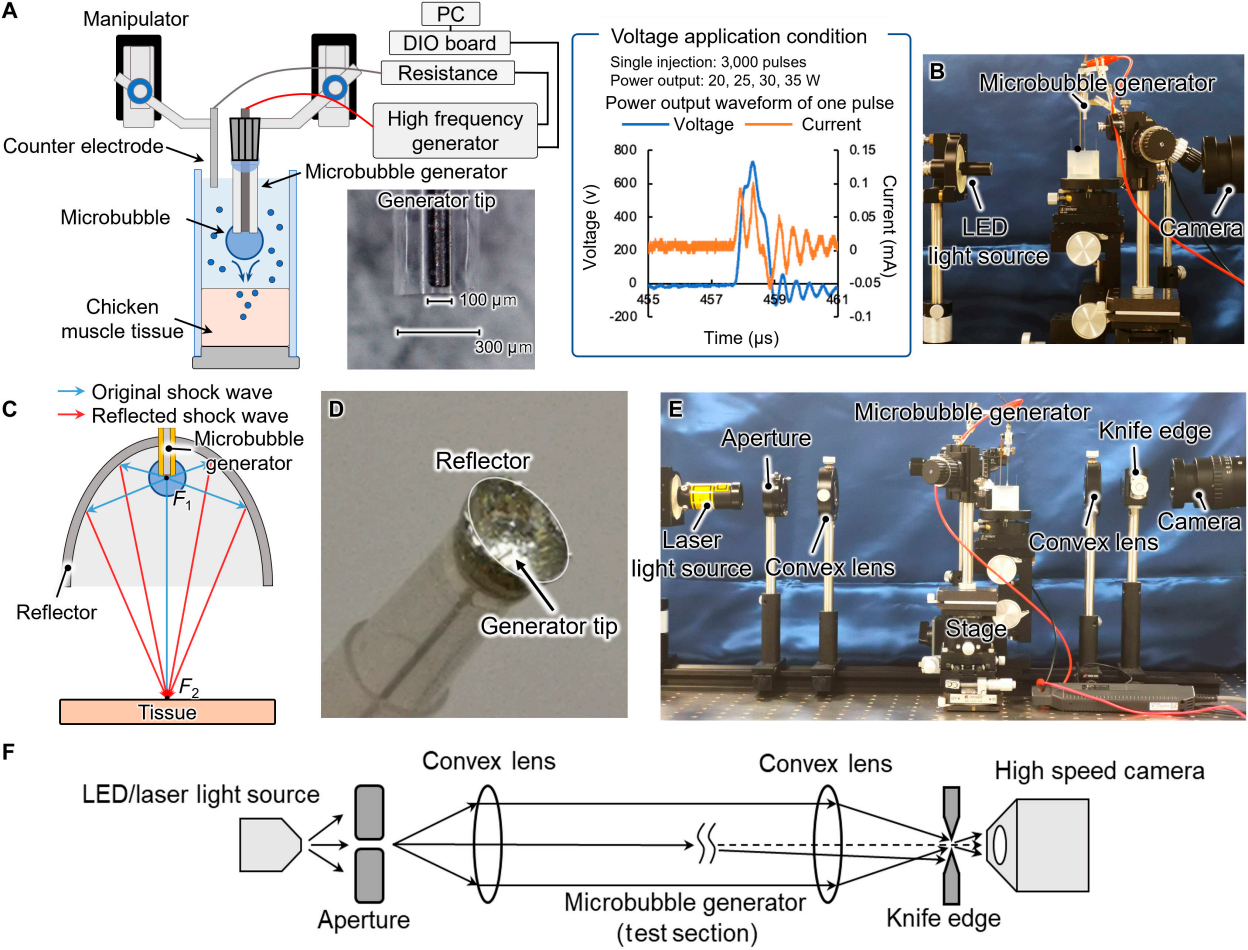
Experimental setup. (A) Schematic of the tissue injection experimental setup. (B) Photograph of a tissue injection experiment. (C) Schematic of the shock wave reflector. (D) Photograph of the microbubble injector integrated with the reflector. (E) Photograph of the schlieren photography experimental setup. (F) Schematic of the optical pathway of the schlieren photography.

Figure 2A is a schematic of the microbubble repetitive mechanical oscillation injection system. The tip of the electrically induced microbubble generator was made of a tungsten wire with a diameter of 100 μm and a Teflon tube with an inner diameter of 150 μm and outer diameter of 300 μm (BEX Co. Ltd., Japan). The tungsten wire was inserted into the Teflon tube, and the distance between the tip of the tungsten wire and the tip of the Teflon tube was 0 to 15 μm. The end of the tungsten wire and Teflon tube was bonded to a metal needle that was connected to a high-frequency power supply (Hyfrecator 2000, CONMED Co. Ltd., USA). The high-frequency power supply was connected to a resistance and counter electrode. The high-frequency power supply supplied 600 pulses in a period of 0.018 s. One pulse is shown in the figure. The reagent was a mixture of fluorescent beads (Fluoro-Max, Thermo Scientific Co. Ltd., USA) with a diameter of 2.1 μm in 0.9% NaCl solution. Manipulators were used to control the height of the microbubble generator. Chicken muscle tissue was used as the target of the reagent introduction. The reagent injection was in a case whose walls were the glass slide. A high-speed camera (HPV-X2, SHIMADZU Co. Ltd., Japan) was used to observe the distance from the microbubble generator tip to the chicken muscle tissue surface, which was set at 10 mm. The experimental setup is shown in Fig. 2B. The high-frequency power supply supplied 5 sets of pulses (i.e., sets of 600 pulses/0.018 s with intervals between sets of 4 s) to the microbubble generator for perforation of the tissue and injection of reagent into the tissue. The experiment was performed at power of 20, 25, 30, and 35 W and with no injection as the control. When a microbubble was generated and the tissue was perforated, the fluorescent beads were delivered into the tissue along with the solution and enclosed by the microbubble-induced slit. After injection, the tissue surface was cleaned under running deionized water. A photograph of the top of the injected tissue wound was taken using a stereo microscope (VHX-7000, KEYENCE Co. Ltd., Japan). The side of the tissue was placed against the objective lens of the microscope. The fluorescence from the cross section of the tissue was imaged under a fluorescence phase-contrast microscope (wavelength of excitation light: 450 to 490 μm, wavelength of emission light: 520 μm; TI-DH, Nikon Co. Ltd., Japan).

The reflector was made from tin owing to its high acoustic impedance relative to that of saline. Reflection and transmission occur when a shock wave conducts from one medium to another. The ratio of reflection to transmission is referred to as the reflectance *R*, which is expressed as [29]

$$R=\frac{A_{2}-A_{1}}{A_{2}+A_{1}}$$ (1)

where *A*_1_ and *A*_2_ are respectively the acoustic impedance of the medium transmitting the shock wave and that of the material at the destination. The equation shows that a greater difference in acoustic impedance between the 2 media corresponds to greater reflectivity. The reflected shock wave is generated when *A*_2_ is greater than *A*_1_, whereas the expansion wave is generated when *A*_2_ is less than *A*_1_. In this experiment, we used shock waves to improve the injection depth, and we thus required a material with an acoustic impedance greater than that of salt water to make the reflector. Here, we chose tin as the material of the reflector because, besides its high acoustic impedance, it is chemically stable and not easily absorbed by tissue and is easy to manufacture. The reflector has a semi-ellipsoidal shape. An ellipsoid has 2 focal points. In our design, we located the microbubble generator tip, which is the point of generation of the microbubble or shock wave, at the first focal point, *F*_1_. The reflector had a focal distance of 10 mm, major axis of 12 mm, and minor axis of 3.32 mm. The design is shown in Fig. 2C. We made the reflector by extruding tin in a mold. We made a hole with a diameter of 0.5 mm at the vertex of the reflector and inserted the injector through this hole. The fabricated reflector is shown in Fig. 2D. We then used the microbubble generator integrated with the reflector for tissue injection. The experimental sequence was the same as that described above.

To verify that the developed reflector reflected the shock wave, we imaged the shock wave and reflected shock wave through schlieren photography. The setup of the schlieren photography is shown in Fig. 2E. The light from the laser source becomes parallel at the first convex lens. Part of the light is deflected as it passes through the test section. The deflected light is blocked by the knife edge and captured by the high-speed camera. The optical pathway is shown in Fig. 2C.

We evaluated the wound depth and area for distances between the microbubble generator tip and tissue surface of 5, 10, and 20 mm (at a focal distance of 10 mm, power of 25 W, and 5 sets of pulses). We then evaluated the wound depth and area for 3, 5, and 10 sets of pulses (at a focal distance of 10 mm, distance between the microbubble generator tip and tissue surface of 10 mm, and power of 25 W). We finally designed and fabricated reflectors with focal distances of 5 and 20 mm, integrated them with the microbubble generator, and evaluated the wound depth and area (at power of 25 W, 5 sets of pulses, and a distance between the microbubble generator tip and tissue surface of 10 mm).

In this study, we used the OriginPro 2024 to do the statistical analysis. Results were from 5 independent runs of the experiment. The differences of each experiment were examined using one-way repeated-measures analysis of variance (ANOVA), which was based on the normality test results. A Tukey test was utilized as the post hoc analysis method. Injection depth and wound area results were graphically represented utilizing the box charts with boxes determined by the 25th and 75th percentiles. A *P* value of <0.05 was considered statistically substantial.

## Results and Discussion

**Fig. 3.**
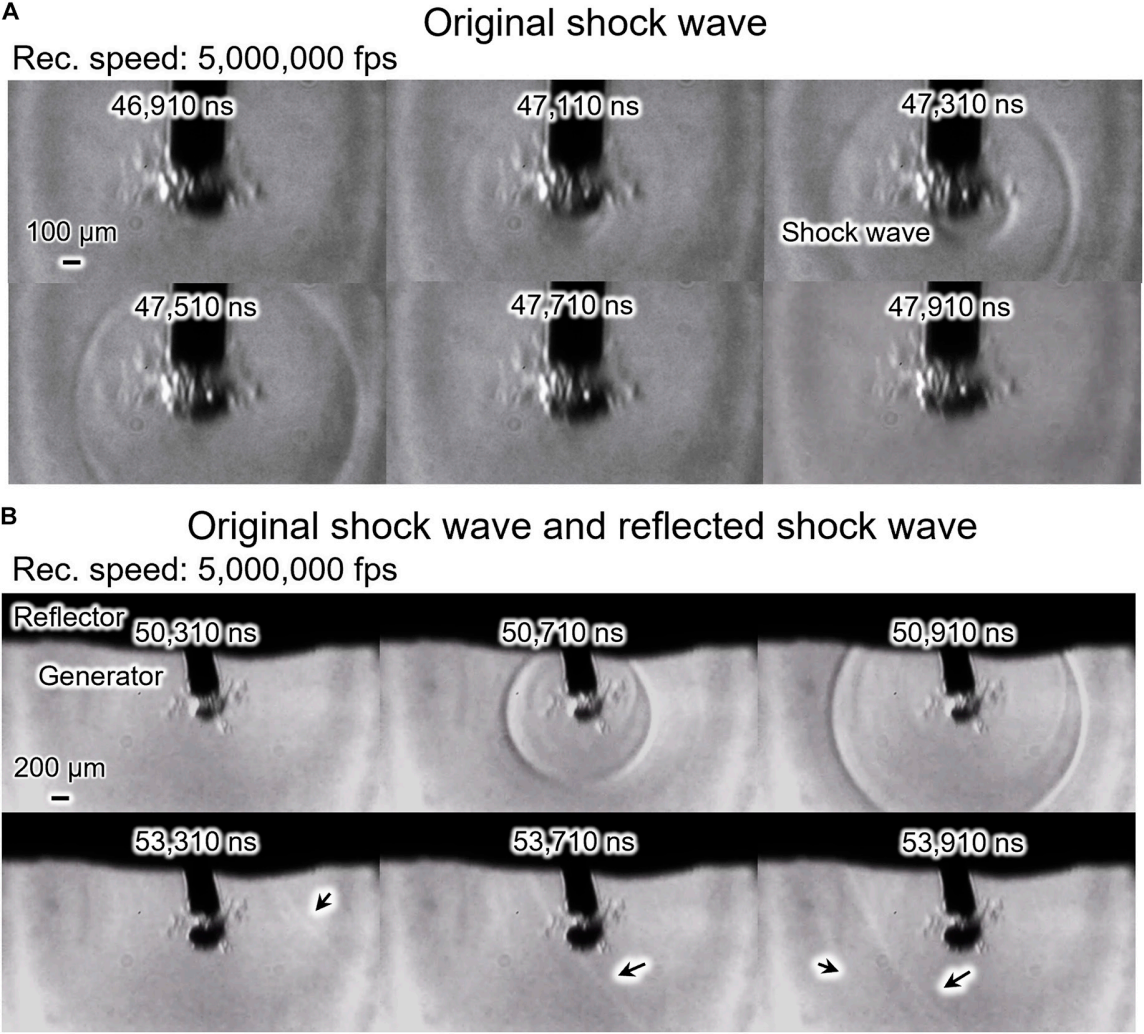
Shock wave schlieren photography. (A) Shock wave generated from microbubble collapse and (B) shock wave generated by microbubble collapse and reflected by the reflector, where reflected shock waves are indicated by black arrows.

Figure 3 shows the schlieren photography of the generation and reflection of a shock wave. Figure 3A shows the generation of the shock wave. The video of a shock wave generated by a microbubble collapse is shown in Movie S2. The microbubble contracted to a minimum volume and generated a shock wave at 47,110 μs (the time elapsed from the voltage application). The shock wave transmitted in all directions. The shock wave is clearly seen at 47,310 and 47,510 ns. Figure 3B shows the shock wave generated from microbubble collapse and reflected by the reflector. Movie S3 shows a shock wave generated by a microbubble collapse and reflected by the proposed reflector. At 50,710 and 50,910 ns, the shock wave from microbubble collapse is seen. At 53,310, 53,710, and 53,910 ns, the reflected shock wave is seen, as marked by arrows. These observations validate our concept of reflecting the shock wave. Our developed reflector can thus reflect and transmit forward a shock wave and increase the mechanical oscillation. However, the reflected shock wave, as seen in the figure, is less intense than the original shock wave owing to the loss of kinetic energy in the conduction of the shock wave.

**Fig. 4.**
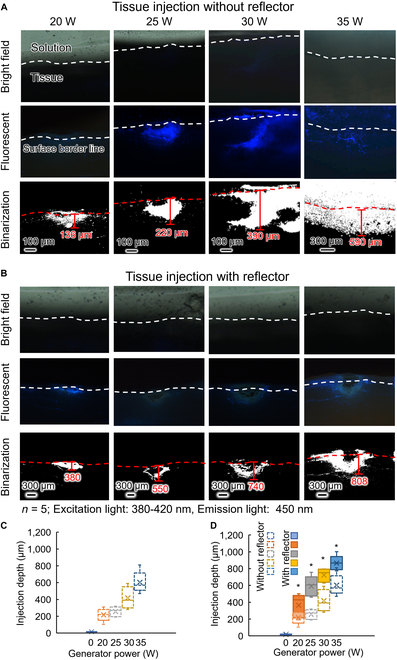
Tissue injection depth. (A and B) Injection depths without/with the reflector, with the first row showing bright-field images, the second row showing fluorescence images of tissue injected with fluorescent beads (used to measure the injection depth), and the third row showing binarized images (white part indicating fluorescence). (C and D) Injection depths at each power without and with reflector. *P < 0.05 versus sample without shock wave reflection (ANOVA using OriginLAB 2024).

Figure 4A shows the cross section of tissue injected with 2.1-μm fluorescence beads through the microbubble injector. The first row of images are bright-field images of the injected tissue, the second row of images are fluorescence images of the tissue injected with fluorescent beads (used to measure the injection depth), and the third row of images are binarized images (where white parts show fluorescence from beads). The tissue surface border line was determined by observation under a microscope and is marked with a white dotted line. There is fluorescence above the border line because it was difficult to make the tissue surface absolutely perpendicular to the cross section, and fluorescent beads remaining on some of the surface were thus also photographed. Injection depths of the samples are noted in the figure. The mean injection depths at power of 0 (control), 20, 25, 30, and 35 W were 20, 220, 250, 380, and 600 μm, respectively. (When the power was lower than 20 W, the injection depth was insufficient for reagent injection, whereas when the power was higher than 35 W, the high voltage posed a risk of electrical shock to the body.) Figure 4C shows that the mean injection depth increased with power. Figure 4B shows the injection result obtained using the reflector, and Fig. 4D shows that the depth of injection increased with increasing power. The mean injection depths at generator power of 0 (control), 20, 25, 30, and 35 W were 20, 350, 570, 690, and 830 μm, respectively. Our reflector was thus effective in increasing the depth of injection.

The electrical energy from the power source was converted into electrical energy dissipated by the system and the kinetic energy of the bubble. The process of a bubble expanding and collapsing generated a microjet and shock wave, and some of the kinetic energy of the microjet and shock wave transferred to the tissue and perforated the tissue. Therefore, as the electrical power increased, the kinetic energy of the microjet and shock wave increased, increasing the depth of injection. As previously stated, the microjet perforates the tissue and the shock wave expands the wound. Following this hypothesis, the result should be a straight line that tapers downward. However, as shown in Fig. 4, the wound was irregularly shaped. This phenomenon is due to the dynamics of the microbubbles. As a microbubble formed and collapsed, it did not disappear completely but repeatedly expanded and contracted. In this process, smaller bubbles formed and remained around the wound. These microbubbles affected the direction of subsequent microbubble movement, giving the wound its irregular pattern.

In the case of using the reflector, the shock wave that made no contribution to perforation was reflected and affected the tissue. As a result, more shock waves impacted the tissue and contributed to perforation. Moreover, its cross-sectional shape was more regular compared to the injection without reflection. We thus consider that our reflector serves to increase the depth of injection by focusing kinetic energy.

**Fig. 5.**
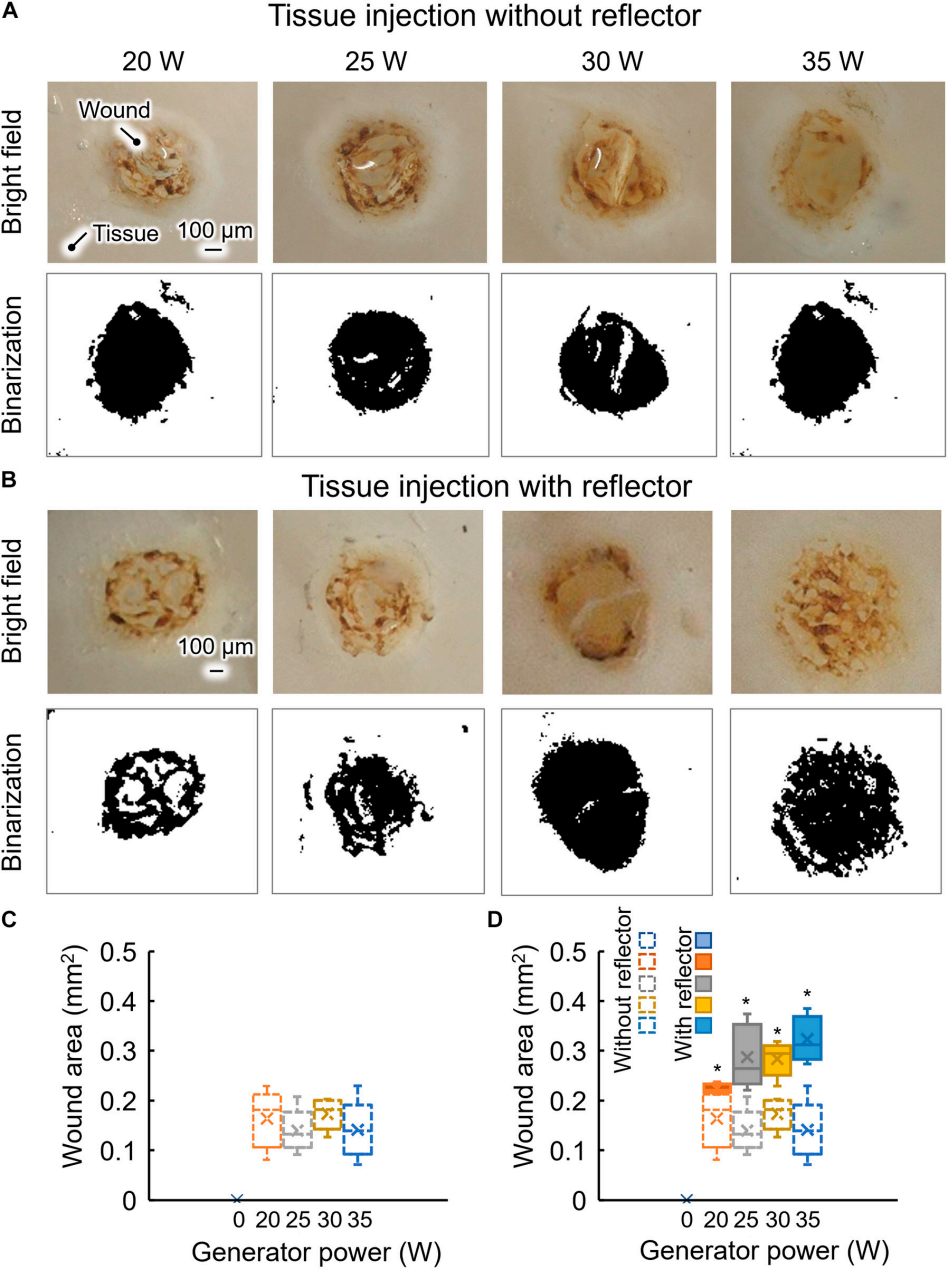
Tissue injection wound area. (A and B) Wound areas without/with reflector, with the first row showing images of the wound and the second row showing binarized images (black part indicating the wound). (C and D) Wound areas at each power without/with the reflector. *P < 0.05 versus sample without shock wave reflection (ANOVA using OriginLAB 2024).

Figure 5 presents images of the perforated wounds, binarized images, and the wound areas for (Fig. 5A) microbubble injection and (Fig. 5B) shock wave reflection microbubble injection. It is seen that the tissue was perforated when we applied the latter injection method. Figure 5C shows the wound area for microbubble injection at different power. The mean areas at power of 20, 25, 30, and 35 W were 0.16, 0.13, 0.17, and 0.14 mm^2^, respectively. There was no substantial difference in the wound area between different values of power. Figure 5D shows the wound area for microbubble injection (no color) and shock wave reflection microbubble injection (colored). The mean areas at power of 20, 25, 30, and 35 were 0.22, 0.29, 0.28, and 0.32 mm^2^, respectively. There was no substantial difference in the wound area between different values of power. The wound area for shock wave reflection was substantial larger than that for microbubble injection at each power. Thus, shock wave reflection increased not only the injection depth but also the wound area. Although the use of the shock wave reflector increases the injection depth, allowing the drug to reach a deeper tissue layer, the larger wound area is not conducive to the drug remaining in the tissue. Therefore, in future work, we will optimize the shock wave reflection concept and suppress the increase in the wound area while increasing the injection depth.

**Fig. 6.**
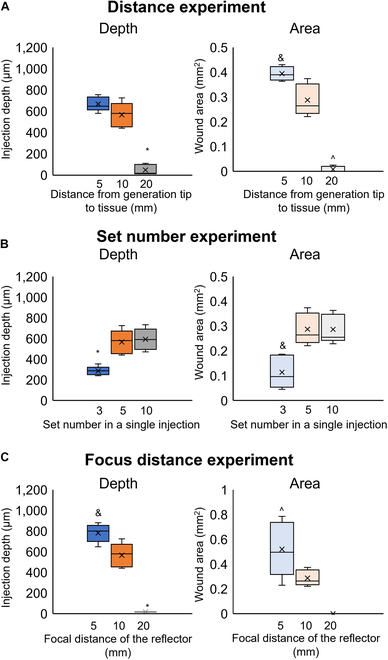
Injection depth and wound area versus (A) the distance from the microbubble generator tip to the tissue surface (*, &, and ^: *P* < 0.05 versus sample at a distance of 10 mm), (B) number of sets of pulses (* and &: *P* < 0.05 versus sample with 5 sets), and (C) focal distance (*, &, and ^: *P* < 0.05 versus sample with a focal distance of 10 mm) (ANOVA using OriginLAB 2024).

Figure 6 presents the injection depth and wound area of tissue for different conditions of shock wave focusing injection. The control conditions were a focal distance of the reflector of 10 mm, distance from the microbubble generator tip to the tissue surface of 10 mm, power of 25 W, and 5 sets of electrical pulses. In the experiments, we changed the conditions of the distance from the microbubble generator tip to the tissue surface and the number of sets of applied electrical pulses. In the experiment in which the reflector focal distance was changed, the distance from the microbubble generator tip to the tissue surface was the focal distance.

Figure 6A shows the injection depth and wound area for distances from the microbubble generator tip to the tissue surface of 5, 10, and 20 mm. The mean injection depths at distances of 5, 10, and 20 mm were 670, 570, and 50 μm, respectively. There was no substantial difference in the mean injection depth between distances of 5 and 10 mm. When distance was 20 mm, in 5 samples, no injection occurred at 3 samples. The depth of last 2 samples was 90 and 110 mm. The mean wound areas for distances of 5, 10, and 20 mm were 0.39, 0.29, and 0.008 mm^2^, respectively. The mean wound area was substantial better (by 0.1 mm^2^) at a distance of 5 mm than at a distance of 10 mm. At a distance of 20 mm, only 2 tissue samples showed a wound in 5 samples. The microjet and shock wave decayed with increasing transmission distance. At 5 mm, there was less attenuation of the shock and microjet and therefore stronger pressure applied to the tissue, which increased the injection depth and wound area. At 20 mm, the kinetic energy of the microjet and shock wave was more attenuated than that at 10 mm. The impact of the microjet and shock wave resulted in a low depth of wound or even no injection. The distance from the microbubble generator tip to the tissue surface thus correlated negatively with the injection depth/wound area.

Figure 6B shows the injection depth and wound area for 3, 5, and 10 sets of pulses. The mean injection depths for 3, 5, and 10 sets of pulses were 290, 570, and 590 μm. The injection depth for 3 sets was substantial lower than that for 5 sets. There was no substantial difference in the injection depth between 5 and 10 sets. The mean wound areas were 0.11, 0.28, and 0.29 mm^2^ for 3, 5, and 10 sets, respectively. There was no substantial difference in the wound areas between the 5 and 10 sets, but the wound area was substantial smaller for 3 sets. In summary, the injection depth and wound area initially increased with the number of sets (i.e., with the number of oscillations) to a certain number of sets. However, stimulation from the actuator no longer affected the perforation after the penetration depth and wound area reached certain levels.

Figure 6C shows the injection depth and wound area for reflector focal distances of 5, 10, and 20 mm. There was no perforation for a reflector focal distance of 20 mm. The mean injection depths were 780 and 570 μm, and the mean wound areas were 0.52 and 0.29 mm^2^ for focal distances of 5 and 10 mm, respectively. It is noted that the wound area had a 5.7% standard deviation at a focal distance of 10 mm and a much larger 19.8% standard deviation at a focal distance of 5 mm. At a focal distance of 5 mm, the transmission path of the shock wave was shorter and there was thus less attenuation of the shock wave. This resulted in an impact with higher pressure on the tissue and thus a greater depth of injection. At a focal length of 20 mm, the attenuation of the shock wave and microjet prevented perforation of the tissue. At a focal length of 5 mm, microbubbles remained in the reflector chamber after injection, which affected the pressure transmission and led to the standard deviation.

## Conclusion

We developed a novel needle-free injection method for reagent injection adopting repetitive mechanical oscillation instead of a normally used high-pressure water jet. We developed a shock wave reflection method. From the schlieren photography, we confirmed that the shock wave from microbubble collapse was reflected. Compared to microbubble tissue injection, by utilizing the reflector, the injection depth of shock wave reflection microbubble tissue injection was improved. This demonstrated the potential of our method for needle-free injection. In the future, we will optimize the shock wave reflection method and focus the shock wave to improve the performance of our needle-free injection method.
